# Impact of Proteinase 3 versus Myeloperoxidase Positivity on Risk of End-Stage Renal Disease in ANCA-Associated Glomerulonephritis Stratified by Histological Classification: A Population-Based Cohort Study

**DOI:** 10.1155/2018/3251517

**Published:** 2018-05-09

**Authors:** Vilde Solbakken, Anne-Siri Fismen, Leif Bostad, Rune Bjørneklett

**Affiliations:** ^1^Department of Clinical Medicine, University of Bergen, Bergen, Norway; ^2^Faculty of Health and Social Sciences, Western Norway University of Applied Sciences, Bergen, Norway; ^3^Department of Pathology, Haukeland University Hospital, Bergen, Norway; ^4^Emergency Care Clinic, Haukeland University Hospital, Bergen, Norway

## Abstract

**Background:**

End-stage renal disease (ESRD) risk in patients with antineutrophil cytoplasmic antibody- (ANCA-) associated glomerulonephritis (ANCA-GN) according to ANCA serotype and stratified by histological classification has not been previously investigated.

**Methods:**

Patients from the Norwegian Kidney Biopsy Registry (NKBR) between 1991 and 2012 who had biopsy-verified pauci-immune glomerulonephritis and positive antineutrophil cytoplasmic antibody serology were included. Cases with ESRD during follow-up were identified in the Norwegian Renal Registry. ESRD-free survival with proteinase 3 (PR3) versus myeloperoxidase- (MPO-) ANCA positivity stratified into 4 histological classes was investigated.

**Results:**

Three hundred fifty-eight patients, of whom 87 progressed to ESRD during follow-up, were included. Patients with PR3- as compared to MPO-ANCA were younger (58 versus 64 years, *p* = 0.001), had a higher percentage of males (62 versus 41%, *p* < 0.001), had a lower percentage with a sclerozing glomerulonephritis pattern (4 versus 16%, *p* < 0.001), and had a significantly higher cumulative ESRD-free survival (90 versus 80%, *p* = 0.007) at 1-year follow-up. No significant differences in cumulative ESRD-free survival with PR3- as compared to MPO-ANCA were observed by histological stratification.

**Conclusion:**

Advanced glomerular sclerosis is found more frequently in patients with MPO-ANCA, explaining the higher risk of ESRD. ANCA serotypes have no impact on prognosis of patients with similar histological findings.

## 1. Introduction

Antineutrophil cytoplasmic antibody- (ANCA-) associated vasculitis (AAV) typically affecting small vessels and kidney involvement in the form of pauci-immune glomerulonephritis (ANCA-GN) is common [1]. Even with modern treatment, AAV is associated with significant morbidity and mortality; moreover, in patients with ANCA-GN, progression to end-stage renal disease (ESRD) resulting in the need for chronic dialysis treatment or kidney transplantation is a particular concern [2]. Although ANCA-GN from a clinical point of view usually is regarded as one disease, it is associated with 2 different autoantibodies, proteinase 3 (PR3) and myeloperoxidase (MPO). A number of differences between patients with PR3- as compared to MPO-AAV have been described [3]. Differing genetic associations, [4, 5] as well as substantial sex differences, have been found [6, 7]. In some studies, worse baseline renal function in patients with MPO- as compared to PR3-ANCA-GN has been reported [8, 9]. The histopathological findings in ANCA-GN are qualitatively similar, irrespective of ANCA type [10, 11]. However, a few studies have noticed quantitative differences in the form of a higher percentage of normal glomeruli in PR3-ANCA-GN and more fibrotic changes in MPO-ANCA-GN [10, 12–14]. Despite a higher risk of relapse [15–17], renal prognosis has often been found better in PR3- than in MPO-AAGN [18–20]. However, it is unclear whether the poorer prognoses associated with MPO- as compared to PR3-ANCA-GN are caused by more advanced disease at baseline [7, 8, 21–24] or due to differences in the nature of inflammation associated with these 2 different autoantibody serotypes [25–27].

The histological classification of patients with ANCA-GN into 4 groups, *focal (≥50% normal glomeruli)*, *crescentic (≥50% cellular crescents)*, *mixed (<50% normal, <50% crescentic, and <50% globally sclerotic glomeruli)*, and *sclerotic (≥50% globally sclerotic glomeruli)*, is useful for stratifying patients according to the risk of progression to ESRD [7, 20, 28–31]. To our knowledge, data comparing renal disease and patient survival with PR3- as compared to MPO-ANCA-GN, in cases with similar histological classification, have not been previously published. Here, using data from the Norwegian Kidney Biopsy Registry, we have compared baseline characteristics, histological findings, and outcomes stratified for histological classification in patients with ANCA-GN divided by PR3- as compared to MPO-ANCA positivity.

## 2. Material and Methods

This study was approved by the Regional Committee for Medical and Health Research Ethics. Data sources used are based on informed consent.

In the present investigation, we included patients with ANCA-GN from a previous study cohort used by our group [29]. Identification of the study cohort, baseline data, scoring of patients according to the histologic classification model of ANCA-GN, definition of observation period, and endpoints were described in detail earlier and only briefly reviewed here.

All patients with a biopsy containing at least 3 glomeruli and histologically verified ANCA-GN in the Norwegian Kidney Biopsy Registry (NKBR) from 1991 to 2012 were included in the study cohort. The criteria for ANCA-GN were the presence of pauci-immune necrotizing glomerulonephritis and a positive ANCA titer. Baseline data, including sex, age, ANCA serotype (determined by indirect immunofluorescence and/or enzyme linked immunosorbent assay methods), estimated glomerular filtration rate (eGFR), serum albumin, systolic and diastolic blood pressure, and proteinuria were obtained from the NKBR. An experienced renal pathologist (LB) classified all cases according to the ANCA-GN histological classification scheme. The observation period was from the time of biopsy to the first event, ESRD, death, or end of 2012. Furthermore, the observation period was stratified according to the induction (≤1 year after biopsy) and the remission (>1 year after biopsy) phases. The primary endpoint of this study was ESRD, defined as the initiation of chronic renal replacement therapy in the form of dialysis or renal transplantation. Cases with ESRD were identified by the linkage of the study cohort with the Norwegian Renal Registry. The secondary endpoint was death. These were identified by linking the study cohort with the Population Registry of Norway and also used to define a combined endpoint, which consists of patients with ESRD or those who died during follow-up, whichever came first.

First, we analyzed baseline data stratified according to the MPO-/PR3-ANCA serotype. Second, we analyzed 1- and 5-year cumulative ESRD-free survival stratified for MPO and PR3 positivity and specifically for each histological class of ANCA-GN using Kaplan-Meier statistics. Finally, Cox regression statistics was used to calculate the hazard ratio (HR) of ESRD with MPO- versus PR3-ANCA after adjustment for histological class, sex, and age. In these analyses, deaths without ESRD were treated as censored events. The analyses were then repeated using the secondary endpoints: ESRD/death (deaths treated as an event) and deaths (deaths with ESRD included as events). For significance testing, Chi-square test was used with categorical variables, Mann–Whitney *U* test for continuous variables, and Log-rank test for comparisons of survival. SPSS, version 24 was used for statistical analyses.

## 3. Results

Three hundred fifty-eight patients were identified and included in the study cohort. One hundred ninety-three (54%) were PR3-ANCA positive. One hundred eighty-seven (52%) were male, and their mean age was 60 years (SD = 17), mean eGFR was 34 ml/min/1.73 m^2^ (SD = 30), and mean percentage glomeruli without crescents or global sclerosis was 36% (SD = 29). Other baseline characteristics are shown in Table 1. Median observation period was 3.5 years (25th–75th percentiles = 0.6–7.7 years) and the total number of patient years was 1716. During the short follow-up period (≤1 year), 50 (14%) of the patients underwent chronic renal replacement therapy and 47 (13%) patients without ESRD died. Two hundred sixty-one (73%) patients survived >1 year after the diagnosis of ANCA-GN without ESRD, 37 (10%) later progressed to ESRD, 31 (9%) without ESRD died, and 193 (54%) were alive without ESRD by the end of 2012. Forty-eight of 87 patients with ESRD were alive by the end of 2012.

Baseline data stratified according to ANCA serotype are shown in Table 1. A significant sex difference was observed. PR3-ANCA was found in 62% of male and 41% of female patients (*p* < 0.001). Mean age in patients with PR3-ANCA was significantly lower (58 versus 64 years) than that in patients with MPO-ANCA positivity (*p* = 0.001). The “sclerotic” histological class was observed in 16% of the patients with MPO-ANCA as compared to 4% with PR3-ANCA (*p* < 0.001). No other significant differences in baseline characteristics were observed, except for relatively minor differences in serum albumin and diastolic blood pressure. Median observation period with PR3-ANCA was 3.9 years (25th–75th percentiles = 0.8–7.8 years) and 2.8 years (25th–75th percentiles = 0.4–7.3 years) with MPO-ANCA. The total number of patient years was 1016 with PR3- and 701 with MPO-ANCA.

**Figure 1:**
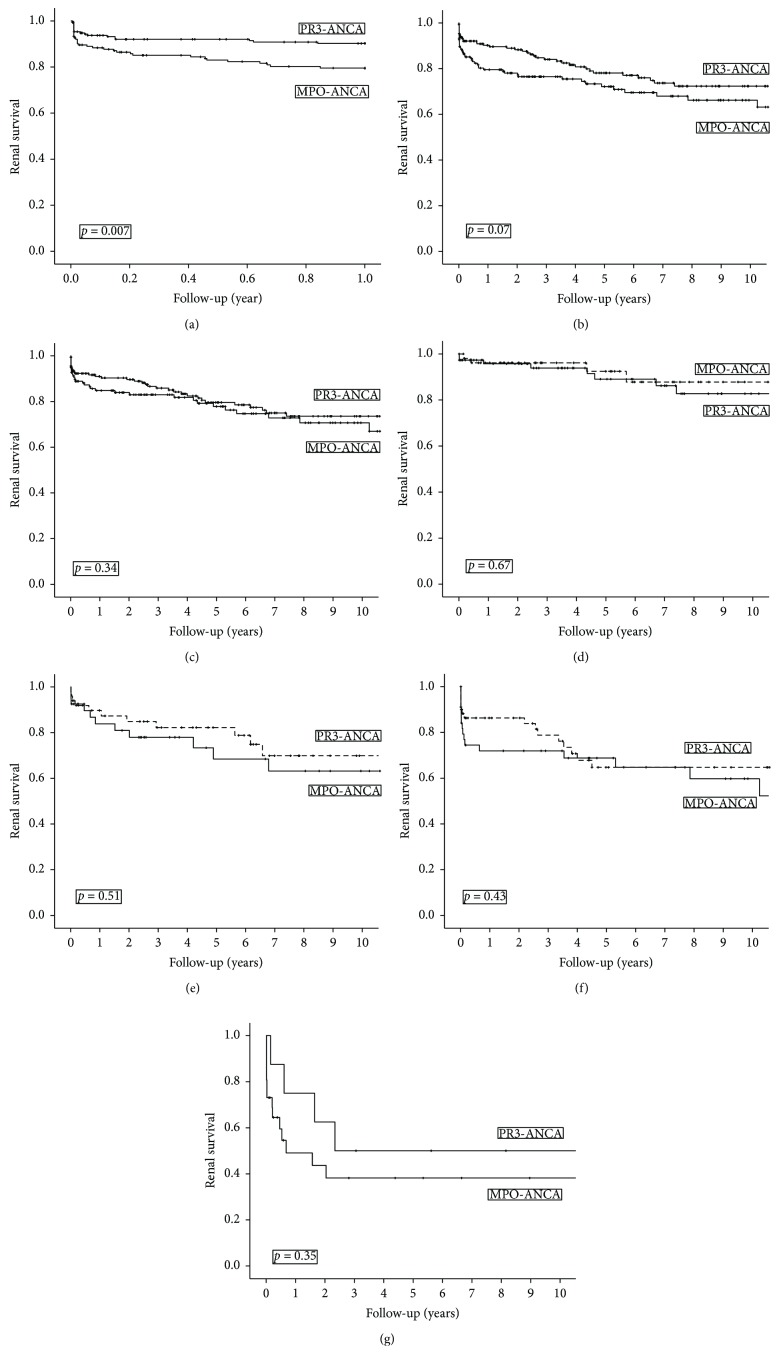
(a) Kaplan-Meier plot demonstrating end-stage renal disease-free survival at 0-1 after diagnosis with MPO- versus PR3-ANCA. (b) Kaplan-Meier plot demonstrating end-stage renal disease-free survival in total observation period with MPO- versus PR3-ANCA. (c) Kaplan-Meier plot demonstrating end-stage renal disease-free survival in total observation period with MPO- versus PR3-ANCA. Cases with sclerotic histology were excluded. (d) Kaplan-Meier plot demonstrating end-stage renal disease-free survival with MPO- versus PR3-ANCA in cases with focal histology. (e) Kaplan-Meier plot demonstrating end-stage renal disease-free survival with MPO- versus PR3-ANCA in cases with mixed histology. (f) Kaplan-Meier plot demonstrating end-stage renal disease-free survival with MPO- versus PR3-ANCA in cases with crescentic histology. (g) Kaplan-Meier plot demonstrating end-stage renal disease-free survival with MPO- versus PR3-ANCA in cases with sclerotic histology.

As demonstrated in Figure 1(a), cumulative survival without ESRD (deaths without ESRD treated as a censoring event) in the 0-1-year observation period was significantly better with PR3- rather than MPO-ANCA (*p* = 0.007). However, when the total observation period was analyzed, the difference was no longer statistically significant (*p* = 0.07, Figure 1(b)). Furthermore, when cases with sclerotic histology were excluded from the analysis, survival without ESRD was basically similar in both ANCA subtypes (*p* = 0.34, Figure 1(c)). One- and 5-year cumulative ESRD-free survival rates with PR3- versus MPO-ANCA stratified according to the histological classification model are shown in Table 2 and Figures 1(d)–1(g). No significant differences were observed during the analysis of the survival rates stratified for histological classification. In the Cox regression analysis, HR for ESRD with MPO- versus PR3-ANCA was 1.30 (0.83–2.02, *p* = 0.25), with adjustments for age, gender, and histological classification.

One- and 5-year cumulative ESRD-free survival (deaths without ESRD treated as an event) with PR3- versus MPO-ANCA stratified according to the histological classification model is shown in Table 3. No significant differences were observed. In the Cox regression analysis, HR for ESRD/death with MPO- versus PR3-ANCA was 1.04 (0.75–1.43, *p* = 0.83), with adjustments for age, sex, and histological classification.

One- and 5-year cumulative patient survival (deaths with ESRD included) with PR3- versus MPO-ANCA stratified according to the histological classification model is shown in Table 4. No significant differences were observed. In the Cox regression analysis, HR for death with MPO- versus PR3-ANCA was 0.77 (0.53–1.13, *p* = 0.18), with adjustment for age, sex, and histological classification.

## 4. Discussion

In this study, we have demonstrated that the risk of ESRD and/or death in patients with ANCA-GN is similar irrespective of PR3- or MPO-ANCA positivity when the histological picture at baseline is similar. The consequence of this finding for clinical practice and future research is that histological classification is more relevant and important than serological classification of ANCA-GN with respect to prognosis. To the best of our knowledge, no study has previously reported ANCA-serotype-specific outcomes of ANCA-GN, stratified by histological classification. Explanation for this surprising lack of data is probably that many studies involved small samples unsuitable for stratified analyses [32]. We hope our findings will encourage researchers to conduct multicenter studies or meta-analyses that will confirm our results.

Our study confirms several previous findings with respect to differences in baseline characteristics of patients with ANCA-GN according to serotype [3]. In a number of studies, the differences in male to female ratio in patients with PR3- as compared to MPO-ANCA GN were identified [3, 6, 7]. A higher mean age in patients with MPO- than PR3-ANCA was also previously described [3]. Similar to our findings, sclerotic histology was significantly more frequent in patients with MPO- than PR3-ANCA in a previous study with patients recruited from Spain and the UK [33]. The probable explanation is the fewer extrarenal symptoms and findings and thus the increased delay in the diagnosis of ANCA-GN in patients with MPO- than with PR3-ANCA. In some studies, patients with MPO-ANCA had higher baseline serum creatinine and a higher percentage of affected glomeruli than those with PR3-ANCA [8–10, 12–14]. We observed the same tendency in the current study. However, differences were not statistically significant. The significantly higher risk of ESRD with MPO- as compared to PR3-ANCA at 1 year of observation is also found in numerous previous studies [3, 18–20]. Here, we can document that this difference in prognosis is solely attributed to a higher fraction with sclerotic histology among patients with MPO- than PR3-ANCA.

The strength of this study is that it included a relatively large population-based study cohort with reliable registration of endpoints from high-quality national registries. However, some limitations must also be considered. We know that the treatment regimen for ANCA-GN in Norway during the study period almost exclusively consisted of cyclophosphamide and steroids, with azathioprine substituting for cyclophosphamide for maintenance treatment after 2003 [19, 29, 34]. However, we do not know the specific treatment plans of individual patients. In a previous study using the same patient cohort, we demonstrated that outcomes were in line with those reported in other studies, indicating that treatment followed acceptable international standards [29]. Another limitation is the lack of data regarding extrarenal vasculitis manifestations. Such data are not registered in the NKBR, which focuses on renal disease.

In conclusion, we have not found ANCA-serotype-specific differences in outcomes of ANCA-GN stratified by histological classification. As a result, we conclude that with respect to prognosis, histological classification is more important than serologic classification in patients with ANCA-GN.

## Figures and Tables

**Table 1:** Baseline characteristics in the total cohort and stratified for MPO- and PR3-ANCA.

Characteristic	All, *n* = 358	MPO-ANCA, *n* = 165	PR3-ANCA, *n* = 193	*p* value
Males (%)	187 (52)	68 (41)	119 (62)	<0.001
Mean age (SD)	60 (17)	64 (15)	58 (18)	0.001
eGFR (SD)	34 (30)	31 (28)	36 (32)	0.07
Serum albumin (SD)	32 (7)	32 (7)	31 (7)	0.04
Systolic blood pressure (SD)	142 (21)	144 (21)	141 (21)	0.08
Diastolic blood pressure (SD)	80 (11)	82 (11)	79 (11)	0.05
Proteinuria (SD)	1.8 (2.0)	1.9 (2.0)	1.7 (2.0)	0.25
Percentage normal glomeruli (SD)	36 (29)	33 (28)	38 (30)	0.07
Focal histology (%)	127 (35)	53 (32)	74 (38)	0.21
Mixed histology (%)	90 (25)	40 (25)	50 (26)	0.82
Crescentic histology (%)	106 (30)	45 (27)	61 (32)	0.32
Sclerotic histology (%)	35 (10)	27 (16)	8 (4)	<0.001

**Table 2:** End-stage renal disease-free survival at 1 and 5 years of follow-up with MPO- and PR3-ANCA, total cohort and stratified according to histological classification.

Characteristic	*N*		ESRD		1 year		5 years		*p* value
	PR3-ANCA	MPO-ANCA	PR3-ANCA	MPO-ANCA	PR3-ANCA	MPO-ANCA	PR3-ANCA	MPO-ANCA	
All	193	165	41	46	90%	80%	78%	72%	0.07
Focal	74	53	8	4	96%	96%	89%	92%	0.67
Mixed	50	40	11	11	90%	84%	82%	69%	0.51
Crescentic	61	45	18	17	86%	72%	65%	69%	0.43
Sclerotic	8	27	4	14	75%	49%	50%	38%	0.35

**Table 3:** Overall and end-stage renal disease-free survival at 1 and 5 years of follow-up with MPO- and PR3-ANCA, total cohort and stratified according to histological classification.

Characteristic	*N*		ESRD/deaths		1 year		5 years		*p* value
	PR3-ANCA	MPO-ANCA	PR3-ANCA	MPO-ANCA	PR3-ANCA	MPO-ANCA	PR3-ANCA	MPO-ANCA	
All	193	165	84	81	76%	69%	62%	55%	0.13
Focal	74	53	25	16	84%	87%	72%	71%	0.95
Mixed	50	40	19	19	80%	75%	73%	53%	0.53
Crescentic	61	45	36	27	64%	62%	43%	52%	0.92
Sclerotic	8	27	4	19	75%	37%	50%	30%	0.16

**Table 4:** Patient survival at 1 and 5 years of follow-up with MPO- and PR3-ANCA, total cohort and stratified according to histological classification.

Characteristic	*N*		Deaths		1 year		5 years		*p* value
	PR3-ANCA	MPO-ANCA	PR3-ANCA	MPO-ANCA	PR3-ANCA	MPO-ANCA	PR3-ANCA	MPO-ANCA	
All	193	165	62	55	84%	85%	70%	74%	0.64
Focal	74	53	19	12	86%	91%	78%	78%	0.97
Mixed	50	40	17	12	86%	82%	72%	74%	0.70
Crescentic	61	45	24	19	77%	87%	60%	76%	1.00
Sclerotic	8	27	2	12	100%	78%	70%	65%	0.37

## Data Availability

An anonymized version of the data file is available from the corresponding author upon request.
